# Impact of PKC-MAPK Signaling on Cardiac Sympathetic Overactivation in Type-2 Diabetes Mellitus

**DOI:** 10.3390/ijms27020723

**Published:** 2026-01-10

**Authors:** Jaswinder Singh, Afia Saabea Owusu Konadu, Yu Li, Boris Shabaltiy, Yu-Long Li

**Affiliations:** Department of Emergency Medicine, University of Nebraska Medical Center, Omaha, NE 68198, USA; jsingh@unmc.edu (J.S.); aowusukonadu@unmc.edu (A.S.O.K.); bshabaltiy@unmc.edu (B.S.)

**Keywords:** cell signaling, satellite glial cells, stellate ganglion, cardiac postganglionic sympathetic neuron, diabetes mellitus

## Abstract

Type-2 Diabetes Mellitus (T2DM) is related to cardiac arrhythmias. The stellate ganglion (SG), part of the sympathetic nervous system, regulates heart function. Within the SG, satellite glial cells (SGCs) have gap junction channels (Cx43). Increased Cx43 permeability induces SGC depolarization and activates the PKC-MAPK14-ADAM17 signaling pathway, releasing some endogenous factors that stimulate nearby cardiac postganglionic sympathetic neurons (CPSN). This study investigated the activation of the PKC-MAPK14-ADAM17 signaling pathway in T2DM SGs and SGCs as a novel mechanism of sympathetic overactivation. A total of 56 Sprague-Dawley rats were randomly assigned to sham and T2DM groups, and T2DM was induced using a high-fat diet combined with low-dose streptozotocin. Real-time RT-PCR, Western blot, and ELISA quantified mRNA/protein expression and enzymatic activity. The patch clamp technique assessed neuronal voltage-gated Ca^2+^ currents and action potentials, while electrophysiological recording measured cardiac sympathetic nerve activity (CSNA). T2DM rats exhibited marked upregulation of MAPK14, PKC-α, and ADAM17 mRNA/protein in the SG, alongside elevated enzymatic activities of PKC and ADAM17. T2DM also increased Ca^2+^ currents and neuronal excitability in the CPSN and induced the elevation of the CSNA. Upregulated PKC-MAPK-ADAM17 signaling in the SG might contribute to cardiac sympathetic overactivation in T2DM rats by enhancing the cell excitability of the CPSN.

## 1. Introduction

Type 2 diabetes mellitus (T2DM) is a pervasive global health burden, currently afflicting approximately 590 million individuals worldwide, a number projected to escalate to 853 million by 2050 [1]. T2DM is widely recognized as a multifaceted metabolic disorder, arising from mounting insulin resistance coupled with pancreatic β-cell dysfunction. Although macrovascular complications are well characterized, cardiac autonomic imbalance, particularly heightened sympathetic activation, remains underappreciated within diabetic cohorts [2,3]. The sympathetic nervous system exerts a profound regulatory influence over cardiac function, with the stellate ganglion (SG) serving as the principal efferent hub mediating sympathetic input to the myocardium [4,5,6]. These bilateral neuronal structures mediate most cardiac sympathetic output. Pathological alterations in these ganglia can precipitate excessive sympathetic drive, inducing arrhythmogenic potential and elevating cardiovascular risk in T2DM and heart failure [5].

The SGs are composed of abundant sympathetic neuronal cell bodies, nerve fiber networks, and a substantial population of satellite glial cells (SGCs). In the physiological condition, these SGCs encapsulate and provide metabolic, trophic, and regulatory support to sympathetic neurons, actively sustaining the neuronal microenvironment and mediating neuroinflammatory homeostatic activity through their intracellular signaling cascades [7,8,9].

A salient feature of SGCs is their robust expression and permeability of connexin 43 (Cx43), a gap junction protein that facilitates intercellular communication and responds to pathological stress [10]. The activation of protein kinase C (PKC, a pivotal kinase) subsequently phosphorylates an array of downstream effectors, including mitogen-activated protein kinase (MAPK14) [11]. MAPK14 activation is reported to induce the activation of tumor necrosis factor alpha convertase enzyme (TACE/ADAM17) [12,13,14], a metalloprotease being responsible for proteolytic cleavage of precursor proteins, thereby promoting the secretion of macrophage colony stimulating factor (M-CSF) [15,16] and other inflammatory cytokines such as TNF-α and IL-1β to the nearby environment [17]. This surge in inflammatory cytokines can drive the recruitment of immune cells and increase cardiac sympathetic activity, thus contributing to heart disorders in T2DM patients [12,14,18,19]. Despite scattered evidence about SGCs activation and neuroinflammatory responses in the SGs, the precise mechanisms and evidence by which T2DM influences SGC activation are lacking. There is a discernible lack of studies employing T2DM experimental models to elucidate the interplay between SGC activation and inflammatory signaling in heightened cardiac sympathetic activation. We hypothesized that hyperglycemia could activate PKC-α-MAPK14-ADAM17 signaling in the SG, enhancing postganglionic neuronal excitability and CSNA in T2DM, which provides a novel glial mechanism of diabetic sympathetic overexcitation. Therefore, the current study aimed to investigate the correlation between the activation of SGCs within the SGs and cardiac sympathetic overactivation in T2DM.

## 2. Results

### 2.1. Induction of T2DM

A rat T2DM model induced by a high-fat diet (HFD) and a low dose of streptozotocin (STZ) in this study has already been described as a well-established animal model of T2DM [20,21,22,23]. This model mimics the clinical characteristics of T2DM patients, such as hyperglycemia, reduced insulin sensitivity, and hyperlipidemia [20,24,25]. After 12-weeks-post T2DM induction, the level of fasting blood glucose was increased in T2DM rats (*p* = 0.001 vs. sham), while there is no statistical difference in body weight between the two groups (*p* = 0.68, Supplementary Table S1).

### 2.2. Colocalization and Quantification of SGCs in the SGs

**Figure 1:**
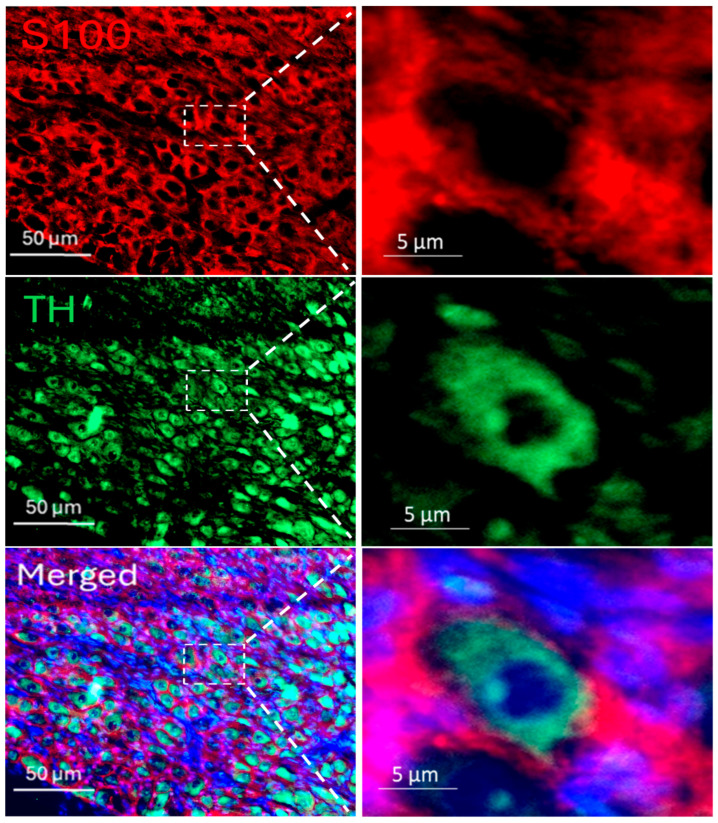
Immunofluorescence staining for the localization of postganglionic sympathetic neurons (green color) and stellate glial cells (SGCs, red color) in a rat SG, which demonstrated that SGCs enveloped postganglionic sympathetic neurons completely in the SG. S100 (S100 calcium-binding protein): a specific marker for SGCs; TH (tyrosine hydroxylase): an adrenergic neuronal marker; DAPI (blue color): a nuclear marker.

**Figure 2:**
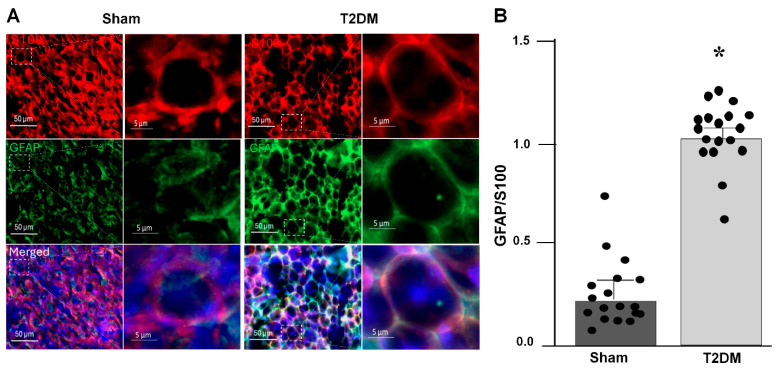
Activation of satellite glial cells (SGCs) in the SGs from T2DM rats, assessed by immunofluorescence staining (IF). (**A**) Representative images of IF showing activated SGCs in sham and T2DM. GFAP (glial fibrillary acidic protein): an activated SGC marker; S100 (S100 calcium-binding protein): a specific marker for SGCs; DAPI: a nuclear marker. (**B**) Quantification of GFAP/S100 was performed by using *n* = 3 animals per group with 6 slices per animal (*p* = 0.001). Each dot represents each slice. GFAP fluorescence intensity was normalized with S100 fluorescence intensity. Data are means ± SEM. An unpaired Student’s *t*-test was used to assess statistical significance. * *p* < 0.05 vs. sham.

Immunofluorescence staining was employed to delineate the spatial distribution of SGCs within the SGs, using tyrosine hydroxylase (TH) as a specific marker for sympathetic neurons and S100 as a specific SGC marker. The staining images revealed that SGCs enveloped postganglionic sympathetic neurons completely in the SGs (Figure 1), thereby providing distinct anatomical localization of SGCs and sympathetic neurons within the SGs. To assess the SGCs activation in T2DM, dual labeling with S100 and glial fibrillary acidic protein (GFAP), a well-recognized marker for activated SGCs [26], was conducted (Figure 2A). Quantitative analysis revealed that the GFAP expression was significantly increased in SGCs from T2DM rats, demonstrating that robust SGC activation under the T2DM condition (Figure 2B).

### 2.3. T2DM Drives Activation of the Kinase Pathway in the SGs

**Figure 3:**
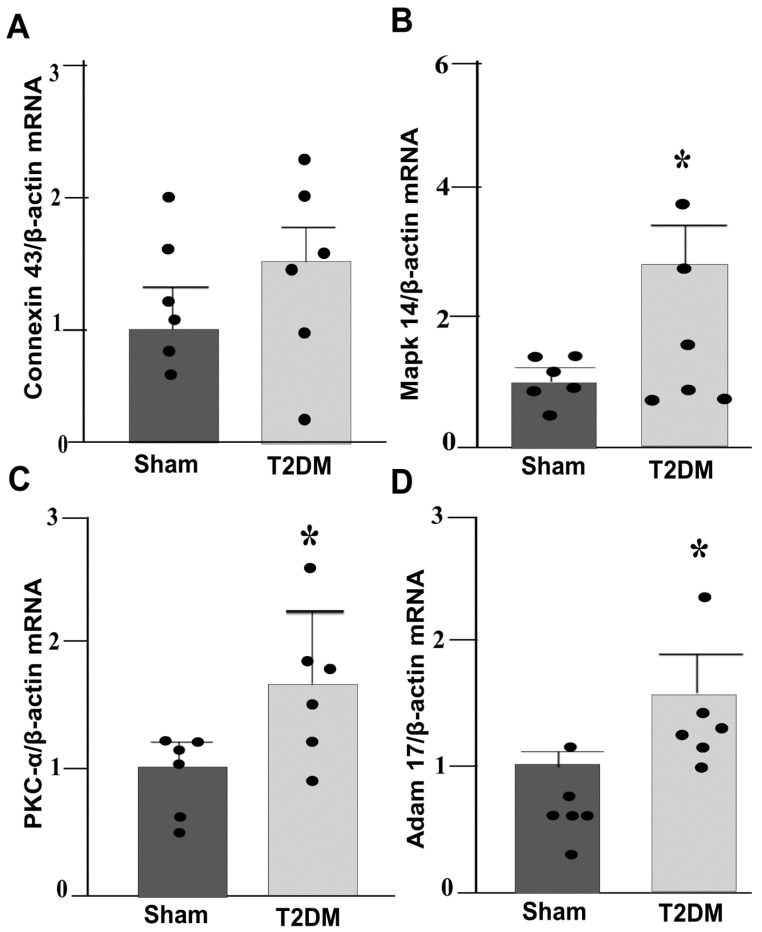
qPCR analysis showing upregulated PKC-α-MAPK14-ADAM17 gene expression in the SGs from T2DM rats: (**A**) Connexin 43 (Cx43, *p* = 0.59); (**B**) MAPK14 (*p* = 0.001); (**C**) PKC-α (*p* = 0.04); (**D**) ADAM17 (*p* = 0.011) mRNA levels in the SGs from sham and T2DM rats (*n* = 6 rats/group). Data are means ± SEM, normalized to housekeeping gene (β-actin). An unpaired Student’s *t*-test was used to assess statistical significance. * *p* < 0.05 vs. sham.

**Figure 4:**
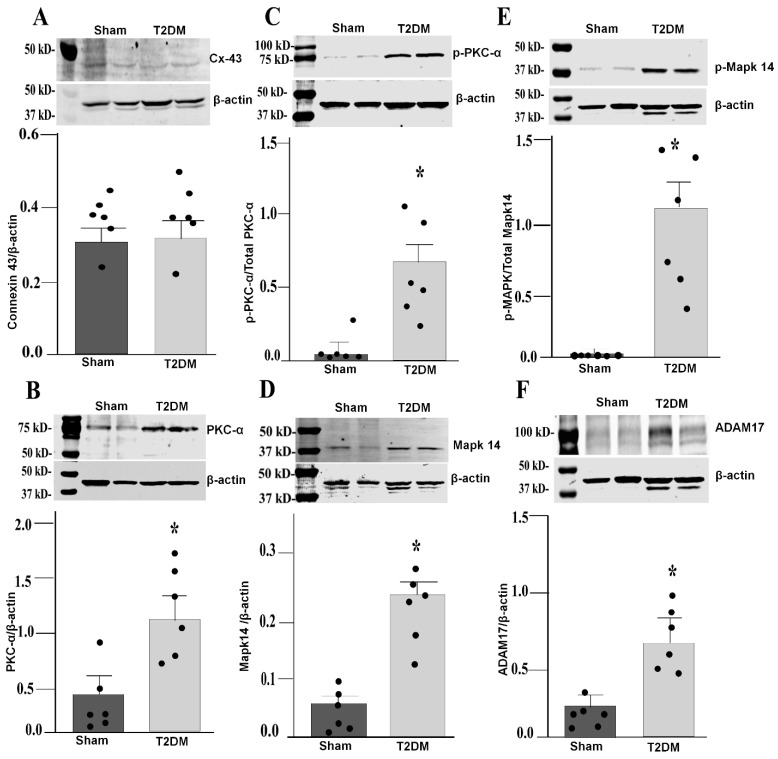
Western blot analysis showing upregulated PKC-α-MAPK14-ADAM17 proteins in the SGs from T2DM rats. Representative images of Western blot and quantification of connexin 43 (*p* = 0.061) (**A**), PKC-α (*p* = 0.007) (**B**), p-PKC-α (*p* = 0.015) (**C**), MAPK14 (*p* = 0.002) (**D**), p-MAPK14 (*p* = 0.014) (**E**), and ADAM17 (*p* = 0.014) (**F**) in the SGs from sham and T2DM rats. *n* = 6 rats/group; data are means ± SEM. An unpaired Student’s *t*-test was used to assess statistical significance. * *p* < 0.05 vs. sham.

**Figure 5:**
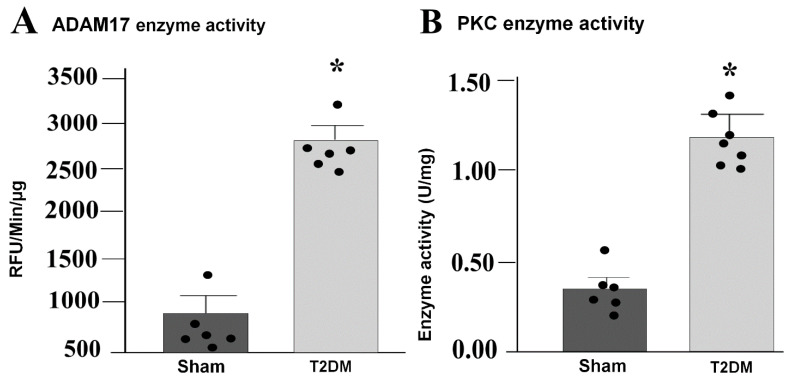
Increased enzymatic activities of ADAM17 and PKC in the SGs from T2DM rats. Enzymatic activity of ADAM17 ((**A**) *p* = 0.001) and PKC ((**B**) *p* = 0.001) in the SGs from sham and T2DM rats, measured by ELISA kits. *n* = 6 rats/group; data are means ± SEM. An unpaired Student’s *t*-test was used to assess statistical significance. * *p* < 0.05 vs. sham.

Integrated analysis of reverse-transcriptase quantitative polymerase chain reaction (RT-qPCR) and Western blot results in the SGs revealed prominent alternations in key signaling pathway in T2DM rats. Quantitative mRNA and corresponding protein expression of PKC-α, MAPK14 and ADAM17 were markedly elevated in the SGs of T2DM, corroborating a pronounced upregulation of molecular mediators at both transcriptional and translational levels (Figure 3 and Figure 4). Additionally, a significant increase in phosphorylated-PKC-α and phophorylated-MAPK14 (an activated state of PKC-α and MAPK14) under the T2DM condition was observed (Figure 4D,F). Furthermore, enzymatic activities of PKC and ADAM17 were also significantly higher in T2DM rats than in sham rats (Figure 5). However, the mRNA and protein expression of Cx43 (a gap junction protein to connect SGCs together) did not exhibit a statistically significant difference between T2DM and sham groups (*p* = 0.59 and 0.061 for Cx43 mRNA and protein, respectively) (Figure 3A and Figure 4A).

### 2.4. Electrophysiological Remodeling of Cardiac Postganglionic Sympathetic Neurons (CPSNs) in T2DM

**Figure 6:**
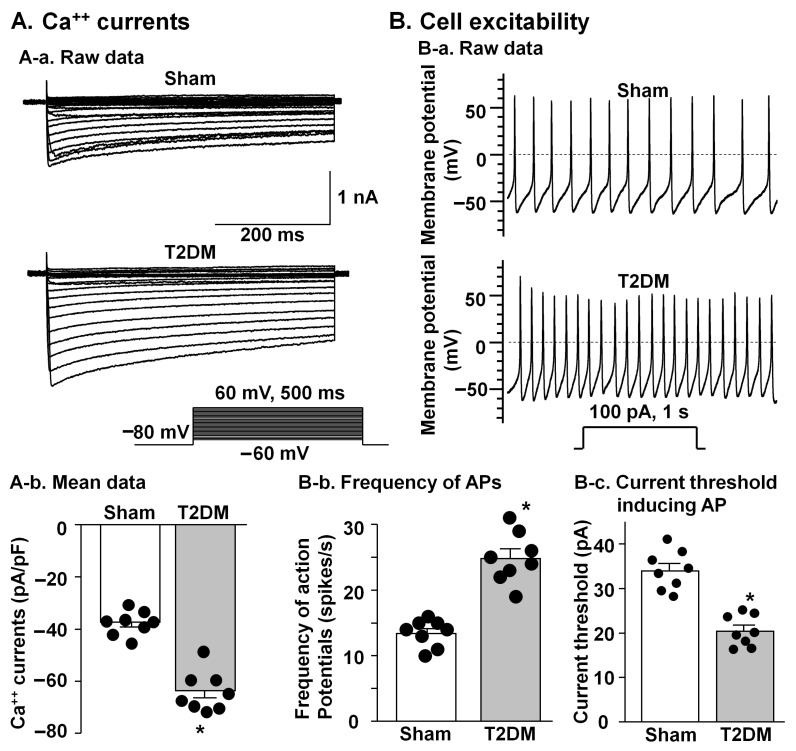
Comparison of voltage-gated Ca^++^ currents and cell excitability in the CPSNs from sham and T2DM rats. (**A**) Original recording (**A-a**) and mean data (**A-b**, *p* = 0.001) of Ca^++^ currents. Mean data for Ca^++^ currents elicited by 500 ms test pulse at 0 mV from holding potential of −80 mV. (**B**) Original recording of action potentials (APs) (**B-a**), frequency of APs (**B-b**, *p* = 0.001), and current threshold inducing APs (**B-c**, *p* = 0.001). *n* = 8 neurons from 4 rats per group; data are means ± SEM. An unpaired Student’s *t*-test was used to assess statistical significance. * *p* < 0.05 vs. sham.

Whole-cell patch clamp recording from isolated CSPNs in the SGs revealed marked functional alterations in T2DM rats. In T2DM neurons, quantitative analyses demonstrated a pronounced augmentation of voltage-gated Ca^2+^ current density, as reflected by increased peak amplitudes (Figure 6A). Assessment of cell excitability showed a significant increase in neuronal firing rates combined with diminished current threshold for induction of action potentials (Figure 6B), indicating that diabetic CPSNs require substantially less depolarizing input to initiate action potential firing and exhibit elevated intrinsic capacity. These electrophysiological adaptations showed a pathological shift toward hyperexcitability and dysregulated Ca^2+^ homeostasis within the cardiac sympathetic network in T2DM.

### 2.5. T2DM Increased the CSNA

**Figure 7:**
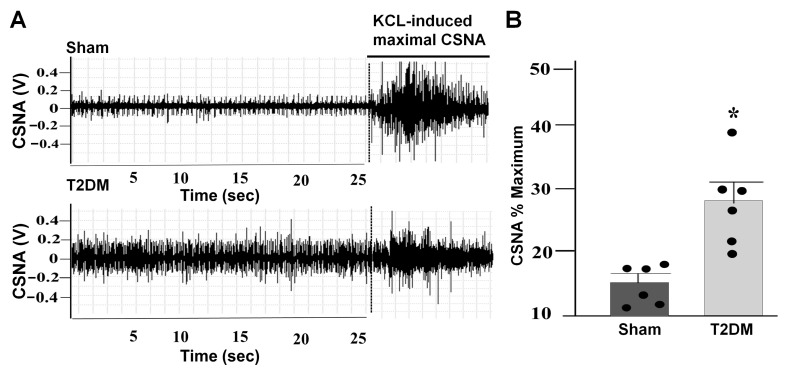
Elevated CSNA in T2DM rats. Representative images (**A**) and quantitative data ((**B**) *p* = 0.01) for the CSNA in sham and T2DM rats. The CSNA was expressed as the percentage of the maximal CSNA induced by KCL. n = 6 rats/group; data are means ± SEM. An unpaired Student’s *t*-test was used to assess statistical significance. * *p* < 0.05 vs. sham.

To investigate the influence of T2DM on cardiac sympathetic activation, the CSNA was recorded in anesthetized rats (Figure 7A). CSNA was found to be significantly elevated in T2DM rats compared to sham rats (*p* = 0.01 vs. sham) (Figure 7B).

## 3. Discussion

The present study confirmed distinct molecular and electrophysiological remodeling within the SGs, including activated SGCs and CPSNs, under the T2DM condition. Persistent hyperglycemia could be associated with pronounced upregulation of the PKC-α-MAPK14-ADAM17 signaling pathway in the SGs, as demonstrated at both mRNA and protein levels. These alterations are consistent with previous studies, which suggest that increased diacylglycerol production and oxidative stress drive the PKC activation, and the latter acts in tandem with MAPK cascade engagement to orchestrate the maladaptive cardiac and vascular remodeling observed in T2DM [27,28,29,30].

### 3.1. The SGs and Cardiac Sympathetic Activation

Cardiovascular autonomic dysfunction, including sympathetic overactivation, is a common complication of patients with T2DM [4,31,32], although the prevalence of cardiovascular autonomic dysfunction in T2DM varies due to different definitions, population characteristics, and diagnostic techniques [33]. Although cardiac sympathetic overactivation is initially beneficial for acute conditions to increase heart rate, cardiac contractility, and conduction velocity, prolonged cardiac sympathetic overactivation has deleterious effects on myocardial structure and function and can lead the progression of cardiac dysfunction in T2DM [34]. In our present study, we also found that the CSNA levels were markedly increased in T2DM rats (Figure 7). In general, the regulation of the CSNA is highly integrated by regulatory circuitry at multiple levels, including cardiac sympathetic afferents, central components, and efferents (the SGs) [35,36,37]. As an important efferent of the cardiac sympathetic nervous system, the CPSNs located in the SGs provide local neural coordination independent of higher brain centers [36,38]. Thus, functional impairment of the CPSNs could be involved in cardiac sympathetic overactivation in T2DM because our present study demonstrated that T2DM induced an increase in the cell excitability of the CPSNs (Figure 6B), which was accompanied by cardiac sympathetic overactivation (Figure 7). Additionally, our present study also showed that voltage-gated Ca^2+^ currents were increased in the CPSNs from T2DM rats compared to sham rats (Figure 6A). A number of patch clamp and imaging studies in animal models of diabetic neuropathy have repeatedly demonstrated abnormal Ca^2+^ influx and increased cytosolic Ca^2+^ accumulation as contributors to neuronal hyperexcitability [39,40,41]. From these data, we postulate that activation of voltage-gated Ca^2+^ channels in the CPSNs contributes to cardiac sympathetic nerve hyperactivity in T2DM.

### 3.2. SGC Activation and Related Signaling Pathway

As a counterpart of astrocytes (a type of glial cells found in the central nervous system), SGCs are located in the peripheral ganglia, including sympathetic, parasympathetic, and sensory ganglia [5,42,43]. Ganglionic SGCs form a network of SGC communication through specific structural proteins (gap junction proteins, such as CX43), which are around ganglionic neural cell bodies to closely contact with each other [5,43,44,45] (Figure 1). This specific neuron-SGC connection provides a structural basis for the connection between ganglionic neurons and SGCs. In particular, our current study demonstrated that the expression of GFAP (a well-known marker for activated SGCs) was higher in the SGCs from T2DM rats than from sham rats (Figure 2), which further confirms that activated SGCs could be involved in cardiac sympathetic overactivation in type 2 diabetes.

Recent studies have provided robust evidence that activated SGCs trigger the secretion of proinflammatory mediators, thereby influencing neuronal function and autonomic balance in pathological conditions such as diabetes [8,42,46]. It has been reported that SGCs activation elevates IL-1β and TNF-α secretion to sensitize nociceptors and sustain pain transmission, thereby establishing glial reactivity as a core driver of cytokine-mediated inflammation with larger applicability to peripheral ganglia, where such signaling pathways may underlie sympathetic dysregulation [42,47]. In addition, exposure to the chemotherapeutic agent oxaliplatin activates the SGCs to heighten IL-6 and TNF-α secretion, establishing cytokine release in the SGs under diabetic hyperglycemia and its impact on neuronal integrity [48,49]. Recently, a study focusing on sensory ganglia SGCs showed that activated SGCs release more IL-6 and TNF-α after a tissue injury to escalate neuronal hypersensitivity and chronic pain, which reflects that diabetes (i.e., hyperglycemia) triggers cytokine-induced sympathetic inflammation [46]. A study using an animal model of myocardial infarction also demonstrated that stellate SGC activation upregulates IL-1β and TNF-α, intensifying cardiac sympathetic hyperinnervation and arrhythmias, while SGC inhibition alleviates the cytokine overdrive and neural overactivity [50], which confirms that SGC targeting could be a viable approach for autonomic dysfunctions akin to those in T2DM.

PKC-α, a member of conventional PKC isoform family, serves as a pivotal signaling mediator molecule in T2DM complications, especially in the SGCs of the SGs where hyperglycemia-driven diacylglycerol accumulation and elevated intracellular Ca^2+^ provide the requisite cofactors for its activation [18,51]. Emerging evidence suggests that enhanced PKC-α activity in SGCs modulates the calcium channel function, neuroinflammatory responses, and autonomic dysregulation in T2DM, potentially via glial release of factors affecting neuronal excitability [18,51,52,53,54,55]. Additionally, therapeutic inhibition of PKC isoforms has shown partial amelioration of neural dysfunctions in diabetic neuropathy models [51,56]. It was reported that activation of PKC-α in the peripheral glia, including SGCs, contributes to glial activation and neuroinflammation, further disrupting autonomic regulation in sensory and sympathetic ganglia under T2DM conditions [48,51].

Our findings demonstrated robust upregulation and activation of both PKC-α and MAPK14 within the SGs under the T2DM condition (Figure 4B,D), aligning with the role of PKC-α in SGC-mediated modulation of Ca^2+^ channel function, neuroinflammation, and autonomic imbalance via glial-derived factors that heighten the neuronal excitability. This T2DM-activated PKC-α intersects with MAPK14 and ADAM17, fostering the proteolytic shedding of proinflammatory cytokines, which amplifies glial reactivity and disrupts sympathetic homeostasis [30,57]. The results of our study are consistent with previous reports of Ca^2+^ dysregulation in diabetic neuropathy, where SGC-derived signals also heighted cytosolic Ca^2+^ and neuronal hyperexcitability [41,58,59,60]. ADAM17 facilitates the proteolytic shedding of cytokines and growth factors, which drives the inflammation and neuronal dysfunction in the metabolic and central nervous system [61,62]. Some studies showed that ADAM17 upregulation amplifies neuroinflammatory responses through the activation of microglia and astrocytes in T2DM [62,63]. In glial cells, ADAM17 facilitates the proteolytic cleavage of proinflammatory cytokines such as TNF-α, IL1-β, and IL6-R, increasing chronic inflammation and neuronal dysfunction in diabetic neuropathy and CNS injury [63,64], or conversely, genetic knockdown of ADAM17 in glial cells suppresses neuroinflammatory cytokine release and reduces glial overactivation [65,66]. PKC inhibition partially reverses these effects in neuropathy models, underscoring glial targeting for the autonomic relief [48,56]. There is no change in Cx43 levels in the present study (Figure 4A), which contrasts with the upregulation of Cx43 in SGCs from diabetic dorsal root ganglia [67]. One possible reasons for this discrepancy is that different tissues and animal models could influence Cx43 alterations. Although total Cx43 levels remained stable, post-translational modifications (e.g., PKC/MAPK-mediated phosphorylation) or gap-junction permeability may contribute to SGC dysfunction in T2DM. For instance, PKC-α mediated phosphorylation at Ser368 closes Cx43 channels without changes in Cx43 expression, enhancing inflammation in diabetic glia [68]. MAPK14/p38 similarly modulated Cx43 gating in neuropathy models, increasing permeability to ATP/cytokines despite stable levels [68]. In the present study, although Cx43 remained stable, PKC-α-MAPK14-ADAM17 signaling is still overactive in the SGs, implying that other endogenous factors and post-translational modifications of Cx43 are involved in the activation of PKC-α-MAPK14-ADAM17 signaling in T2DM.

Although the pathogenesis of cardiac postganglionic sympathetic neuronal over-excitation in T2DM has not been fully elucidated, the role of SGC–neuron crosstalk in the SGs cannot be ignored, based on the specific SGC–neuron structural connection (Figure 1). SGC-derived TNF-α amplifies neuronal action potential firing and norepinephrine release, as reported in SG slices where SGC inhibition attenuates CSNA overdrive [5,50,52]. The SGs from cardiomyopathic patients with arrhythmias showed neuroinflammation, oxidative stress, and SGC activation, indicating an immune-autonomic shift within the SG microenvironment. These pathological features drive excessive CSNA, which provides clinical rationale for SG-targeted therapies in arrhythmic management [8]. Overall, the T2DM-induced SGC activation and excited PKC-α-MAPK14-ADAM17 signaling in SG lysates reported in the present study are considered to contribute to cardiac sympathetic overactivation in T2DM. SGC-dominant PKC-α-MAPK14-ADAM17 signaling emerges as a therapeutic hub to mitigate neuronal remodeling and cardiac sympathetic overactivation in T2DM.

There are some limitations in the present study. First, IF co-staining in future studies is needed to validate SGC specificity, building on current WB/IF localization. Second, considering that we did not perform pharmacological or genetic inhibition of PKC-α, MAPK14, or ADAM17 to test causality in the present study, our future work, incorporating selective blockade of these targets, ideally in both cellular and in vivo models, will be important to definitively establish mechanistic links and to evaluate the therapeutic potential of the signaling pathway inhibition. Third, direct cytokine quantification in future studies will confirm the ADAM17-dependent release of these cytokines in SGCs. Finally, although CSNA hyperactivity implicates arrhythmogenic risk, direct cardiac functional assessments (e.g., ECG-derived QT prolongation, HRV alterations, or programmed stimulation-induced arrhythmia inducibility) are needed. Future studies will evaluate heart rhythm problems and ECG changes after blocking the SG signaling pathway, linking CSNA hyperactivity to cardiac risks.

## 4. Materials and Methods

This study conforms to the guidelines for the Care and Use of Laboratory Animals and was approved by the institutional Animal Care and Use Committee (IACUC, No 18-023-04-FC) at the University of Nebraska Medical Center. We followed our approved IACUC protocol to reduce pain, suffering, and distress and monitor the humane endpoints.

### 4.1. Experimental Design

In the present study, a total of 56 male and female Sprague-Dawley rats (190–210 gm) were housed under standardized conditions with controlled ambient environment of temperature and humidity, maintaining a 12 h light/12 h dark photoperiod. These rats were randomly assigned to sham and T2DM groups. The sham rats were provided with a standard chow diet (including 34% protein, 53% carbohydrate, 13% fat), while T2DM rats were fed with an HFD (15.2% protein, 42.7% carbohydrate, 42% fat; Inotiv Pharmaceutical Company, Indianapolis, IN, USA). After 4 weeks of HFD, a single low dose of STZ (30 mg/kg) was intraperitoneally injected into rats, who continued to be fed on an HFD for up to 12 weeks to induce the T2DM model. All experiments procedures were performed at the end of 12 weeks. Fasting blood glucose over 250 mg/dL is regarded as hyperglycemia. Five rats with less than 250 mg/dL of fasting blood glucose were excluded from the present study (not included in the total animal numbers in the present study). The basic hemodynamic and metabolic characteristics of the rats used in this study are given in Supplementary Table S1. To achieve robust and unbiased data, all animals and tissue samples were blinded during experimentation (including the allocation) and data acquisition until statistical analysis.

### 4.2. Tissue IF

Isolated SGs were fixed in 4% paraformaldehyde prepared in 0.1% PBS to achieve optimal morphological preservation. Fixed tissues were cryoprotected by incubation in 30% (*w*/*v*) sucrose in PBS at 4 °C solution until complete infiltration, then in embedded optimal cutting temperature (O.C.T.) compound (Tissue-Tek O.C.T. Compound, Sakura Finetek, Torrance, CA, USA) for cryosectioning. Serial tissue sections were cut at thicknesses of 10 µm using a cryostat (CM 1950, Leica Biosystems, Nussloch, Germany), maintained at −20 °C, and mounted on superfrost plus glass slides.

Following sectioning, slides were rinsed in PBS for 10 min to remove excess O.C.T.; then, slides were subjected to permeabilization with 0.5% PBS-T (PBS containing triton X-100) for 45 min. Slides were washed three times in PBS (10 min each) to ensure through removal of unbound reagents, followed by blocking with 10% donkey serum for 1 h at room temperature. Tissue sections were incubated with target specific appropriate primary antibodies (see Supplementary Table S2) at 4 °C overnight to facilitate robust antigen–antibody binding. After incubation, slides were washed and exposed to fluorophore-conjugated secondary antibodies and DAPI (4′,6′-diamidino-2-phenylindole) at a final concentration of 1 µg/mL for 1 h at room temperature in dark conditions. Slides were washed three times, air dried, and mounted with fluorescent mounting medium (Flouromount-G, SouthernBiotech, Birmingham, AL, USA). Immunolabeled sections and nuclei were visualized under a Leica fluorescence microscope (Leica Microsystems, Buffalo Grove, IL, USA) equipped with excitation/emission filters. Quantitative analysis was conducted using Adobe Photoshop 2024 (Adobe Systems, San Jose, CA, USA).

### 4.3. RT-qPCR Analysis

Total RNA was isolated from rat SG using Trizol reagent (Cat. No. 15596026, Thermofisher, Walham, MA, USA) in accordance with manufacturer’s protocol. Subsequently, 1000 ng of purified RNA was reverse transcribed into complementary DNA (cDNA) synthesis employing iScript Reverse Transcription Supermix (Cat. No. 1708840, Bio-rad laboratories, Hercules, CA, USA) at 42 °C for 30 min. qPCR was performed utilizing 1 uL of synthesized cDNA as a template in conjugation with iQ SYBR Green Supermix (Cat. No. 1708882, Bio-rad laboratories, Hercules, CA, USA) to detect target amplification specificity (Step one Plus Real Time PCR system, Applied Biosystems, Thermofisher, Waltham, MA, USA). The thermal cycle conditions, as well as the primers sequences used for target gene amplification, are provided in Supplementary Table S3. Relative gene expression levels were quantified by normalizing the target transcript to β-actin and analyzed using the 2^−∆∆Ct^ method.

### 4.4. Western Blot Analysis

For protein expression analysis, rat SGs were promptly excised, rinsed with phosphate-buffered saline (PBS), and stored at −20 °C until analysis. Tissue homogenization was performed in RIPA lysis buffer supplemented with 50 mM Tris-HCL, 150 mM sodium chloride (NaCl), 50 mM sodium fluoride (NaF), 2 mM EDTA, 1 mM Na_3_VO_4_, 1% NP-40, 1% SDS, 1 mM PMSF, and a protease inhibitor cocktail (Cat. No. HY-K0010 MedChemExpress LLC, NJ, USA) using a bead-based homogenizer (Benchmark Scientific Sayreville, NJ, USA). Lysates were incubated on ice for 1 h then centrifuged at 12,000× *g* for 20 min at 4 °C. Supernatants were collected, and protein concentration were quantified by BCA assay (Cat. No. 23225, Pierce BCA Protein Assay Kit, ThermoFisher Scientific, Waltham, MA, USA) to ensure uniform loading across all samples.

For separation, 60 µg of total protein per sample was subjected to electrophoresis on 10% SDS-PAGE gels at 80 V for 20 min and 100 V for 1 h at room temperature. Proteins were transferred onto the polyvinylidene fluoride (PVDF) membrane using a wet transfer method at 100 V for 1.5 h. Membranes were incubated for 1 h at room temperature with intercept blocking buffer (Cat. no. 927-70001, LI-COR, Lincoln, NE, USA).

Blocked membranes were incubated overnight at 4 °C with primary antibody (listed in Supplementary Table S2) on a rotator. After three washes with TBS-T for 10 min at room temperature, blots were incubated for 1 h at room temperature with species-specific fluorescence secondary antibodies. Following the final set of three washes in TBS-T, target bands were visualized using the LI-COR Odyssey DLX imaging system (LI-COR, Lincoln, NE, USA). Densiometric analysis was conducted using ImageJ analysis software (version 1.54m NIH, Bethesda, MA, USA).

### 4.5. Evaluation of Sympathetic Ganglionic Enzymatic Activities

For evaluation of PKC enzyme activity, rat SGs were homogenized in MOPS buffer. The relative kinase activity in lysates was assessed using the PKC kinase activity assay kit (Cat. No. ab139437, Abcam, Cambridge, UK). TACE activity was quantified in SG extracts using the Sensolyte 520 TACE (alpha secretase) activity assay kit (Fluorometric) (Cat. No. AS-72085, AnaSpec Inc., Fremont, CA, USA), following the manufacturer’s instruction. Enzymatic activities were reported in relative fluorescent units (RFU/min).

### 4.6. Isolation of CPSNs in the SGs and Whole Cell Patch Clamp Recording for Ca^2+^ Currents and Action Potentials

After in vivo experiments were performed, bilateral SGs were exposed and removed quickly. The CPSNs were isolated via a two-step enzymatic digestion protocol, as previously described [69,70,71]. Briefly, isolated SGs were placed in ice-cold modified Tyrode’s solution (mM): 140 NaCl, 5 KCL, 10 HEPES, 5 glucose. The SGs were then minced into small pieces with microscissors and incubated with a modified Tyrode’s solution containing 0.1% collagenase and 0.1% trypsin for 30 min at 37 °C. The tissue was then transferred to a modified Tyrode’s solution containing 0.2% collagenase and 0.5% bovine serum albumin for 30 min of incubation at 37 °C. The isolated CPSNs were cultured at 37 °C in a humidified atmosphere of 95% air–5% CO_2_ for 4–8 h before patch clamp experiments.

Voltage-gated Ca^2+^ currents and action potentials were recorded by the whole-cell patch clamp technique using the Axopatch 200B patch clamp amplifier (Axon Instruments, San Jose, CA, USA). Resistance of the patch pipette was 4–6 MΩ when filled with the following solution (in mM): 120 CsCl, 1 CaCl_2_, 40 HEPES, 11 EGTA, 4 MgATP, 0.3 Tris-GTP, 14 creatine phosphate, and 0.1 leupeptin (pH 7.3; 305 mosM). The extracellular solution consisted of (in mM) 140 TEA-Cl, 5 BaCl_2_, 1 MgCl_2_, 10 HEPES, 0.001 TTX, 2 4-AP, and 10 glucose (pH 7.4; 310 mosM). Series resistance of 5–13 MΩ was electronically compensated by 30–80%. Junction potential was calculated to be +7.9 mV using pCLAMP 10.2 software, and all values of membrane potential given throughout were corrected using this value. Current traces were sampled at 10 kHz and filtered at 5 kHz. The holding potential was −80 mV, and current–voltage (I–V) relationships were elicited by 5 mV step increments to potentials between −60 mV and 60 mV for 500 ms. Peak currents were measured for each test potential, and current density was calculated by dividing peak current by cell membrane capacitance (26.9 ± 2.0 pF in sham and 25.6 ± 1.8 pF in T2DM neurons, *p* = 0.643).

In current-clamp experiments, action potentials were elicited by a ramp current injection of 0–100 pA, and the current threshold-inducing action potential was measured at the beginning of the 1st action potential. The action potential frequency was measured in a 1 s current clamp at 100 pA. The patch pipette solution was composed of (in mM) 105 K-aspartate, 20 KCl, 1 CaCl_2_, 5 MgATP, 10 HEPES, 10 EGTA, and 25 glucose (pH 7.2; 320 mOsm/L). The bath solution was composed of (in mM) 140 NaCl, 5.4 KCl, 0.5 MgCl_2_, 2.5 CaCl_2_, 5.5 HEPES, 11 glucose, and 10 sucrose (pH 7.4; 330 mOsm/L). Junction potential was calculated to be +12.3 mV, and membrane potential was corrected using this value. The P-clamp 10.2 program (Axon Instruments, San Jose, CA, USA) was used for data acquisition and analysis. All experiments were performed at room temperature (22–24 °C).

### 4.7. Measurement of the CSNA in Anesthetized Rats

The CSNA was assessed in rats under anesthesia, following the protocols described in previous studies [71,72]. Anesthesia was induced by intraperitoneal administration of 800 mg/kg urethane combined with 40 mg/kg α-chloralose. Following a left thoracotomy, the left cardiac sympathetic nerve was identified, carefully isolated, and placed on a bipolar recording electrode. The nerve signals were amplified, and band-pass filtered to optimize the discrimination of the CSNAs and background noise/movement artifacts. Data acquisition was continuously performed using Labchart software version 7.0 (ADInstruments, Colorado Springs, CO, USA). Throughout the entire procedure, core body temperature was maintained at 37 °C with a heat pad. To analyze the evoked sympathetic responsiveness, potassium chloride (KCL) (3 M, 0.3 mL/100 g) was administered to generate a maximum CSNA. The CSNA was expressed as the percentage of the maximum CSNA.

### 4.8. Statistical Analysis

All data are expressed as means ± SEM. Statistical analyses were conducted using GraphPad Prism version 10.5.1 software (GraphPad Software Inc., La Jolla, CA, USA). Differences between two groups were evaluated using an unpaired Student’s *t*-test after confirming the normal data distribution with the Kolmogorov–Smirov test. A *p*-value of less than 0.05 was considered statistically significant.

## 5. Conclusions

Collectively, these findings, combined with previous studies, indicate that T2DM-induced SGC activation in the SG engages ADAM17 and amplifies cytokine shedding in the SG microenvironment, thereby sensitizing adjacent CPSNs, and the excitation of the latter could be involved in cardiac sympathetic overactivation in T2DM.

## Data Availability

The original contributions presented in this study are included in the article/Supplementary Materials. Further inquiries can be directed to the corresponding author.

## Supplementary Materials

The following supporting information can be downloaded at https://www.mdpi.com/article/10.3390/ijms27020723/s1.

## References

[1] Atlas D. (2025). International diabetes federation. IDF Diabetes Atlas.

[2] De Rosa S., Arcidiacono B., Chiefari E., Brunetti A., Indolfi C., Foti D.P. (2018). Type 2 diabetes mellitus and cardiovascular disease: Genetic and epigenetic links. Front. Endocrinol..

[3] Sudo S.Z., Montagnoli T.L., Rocha B.d.S., Santos A.D., De Sá M.P., Zapata-Sudo G. (2022). Diabetes-induced cardiac autonomic neuropathy: Impact on heart function and prognosis. Biomedicines.

[4] Kanagala A., Harsoda J. (2024). Sympathetic overactivity and parasympathetic impairment in type 2 diabetes: An analysis of cardiovascular autonomic functions. Cureus.

[5] Li Y.-L. (2022). Stellate ganglia and cardiac sympathetic overactivation in heart failure. Int. J. Mol. Sci..

[6] Huggett R.J., Scott E.M., Gilbey S.G., Stoker J.B., Mackintosh A.F., Mary D.A. (2003). Impact of type 2 diabetes mellitus on sympathetic neural mechanisms in hypertension. Circulation.

[7] Mapps A.A., Boehm E., Beier C., Keenan W.T., Langel J., Liu M., Thomsen M.B., Hattar S., Zhao H., Tampakakis E. (2022). Satellite glia modulate sympathetic neuron survival, activity, and autonomic function. Elife.

[8] Ajijola O.A., Hoover D.B., Simerly T.M., Brown T.C., Yanagawa J., Biniwale R.M., Lee J.M., Sadeghi A., Khanlou N., Ardell J.L. (2017). Inflammation, oxidative stress, and glial cell activation characterize stellate ganglia from humans with electrical storm. JCI Insight.

[9] Hanani M. (2024). Satellite glial cells in human disease. Cells.

[10] Sáez J., Contreras-Duarte S., Gómez G., Labra V., Santibañez C., Gajardo-Gómez R., Avendaño B., Díaz E., Montero T., Velarde V. (2018). Connexin 43 hemichannel activity promoted by pro-inflammatory cytokines and high glucose alters endothelial cell function. Front. Immunol..

[11] Igarashi M., Wakasaki H., Takahara N., Ishii H., Jiang Z.-Y., Yamauchi T., Kuboki K., Meier M., Rhodes C.J., King G.L. (1999). Glucose or diabetes activates p38 mitogen-activated protein kinase via different pathways. J. Clin. Investig..

[12] Xu P., Liu J., Sakaki-Yumoto M., Derynck R. (2012). TACE activation by MAPK-mediated regulation of cell surface dimerization and TIMP3 association. Sci. Signal..

[13] Yu Y., Cao Y., Bell B., Chen X., Weiss R.M., Felder R.B., Wei S.-G. (2019). Brain TACE (tumor necrosis factor-α–converting enzyme) contributes to sympathetic excitation in heart failure rats. Hypertension.

[14] Saad M.I., Jenkins B.J. (2024). The protease ADAM17 at the crossroads of disease: Revisiting its significance in inflammation, cancer, and beyond. FEBS J..

[15] Schumacher N., Rose-John S. (2022). ADAM17 orchestrates Interleukin-6, TNFα and EGF-R signaling in inflammation and cancer. Biochim. Biophys. Acta (BBA)-Mol. Cell Res..

[16] Adu-Amankwaah J., Adzika G.K., Adekunle A.O., Ndzie Noah M.L., Mprah R., Bushi A., Akhter N., Huang F., Xu Y., Adzraku S.Y. (2021). ADAM17, a key player of cardiac inflammation and fibrosis in heart failure development during chronic catecholamine stress. Front. Cell Dev. Biol..

[17] Kawai T., Elliott K.J., Scalia R., Eguchi S. (2021). Contribution of ADAM17 and related ADAMs in cardiovascular diseases. Cell. Mol. Life Sci..

[18] Pan D., Xu L., Guo M. (2022). The role of protein kinase C in diabetic microvascular complications. Front. Endocrinol..

[19] Wang X., Liu R., Liu D. (2025). The role of the MAPK signaling pathway in cardiovascular disease: Pathophysiological mechanisms and clinical therapy. Int. J. Mol. Sci..

[20] Liu J., Tu H., Zheng H., Zhang L., Tran T.P., Muelleman R.L., Li Y.-L. (2012). Alterations of calcium channels and cell excitability in intracardiac ganglion neurons from type 2 diabetic rats. Am. J. Physiol.-Cell Physiol..

[21] Hu W., Zhang D., Tu H., Li Y.-L. (2021). Reduced cell excitability of cardiac postganglionic parasympathetic neurons correlates with myocardial infarction-induced fatal ventricular arrhythmias in type 2 diabetes mellitus. Front. Neurosci..

[22] Ghasemi A., Jeddi S. (2023). Streptozotocin as a tool for induction of rat models of diabetes: A practical guide. EXCLI J..

[23] Guo X.-X., Wang Y., Wang K., Ji B.-P., Zhou F. (2018). Stability of a type 2 diabetes rat model induced by high-fat diet feeding with low-dose streptozotocin injection. J. Zhejiang Univ.-Sci. B.

[24] Zhang M., Lv X.-Y., Li J., Xu Z.-G., Chen L. (2008). The characterization of high-fat diet and multiple low-dose streptozotocin induced type 2 diabetes rat model. J. Diabetes Res..

[25] Skovsø S. (2014). Modeling type 2 diabetes in rats using high fat diet and streptozotocin. J. Diabetes Investig..

[26] Kamada J., Hamanaka T., Oshimo A., Sato H., Nishii T., Fujita M., Makiguchi Y., Tanaka M., Aoyagi K., Nojima H. (2024). Glial fibrillary acidic protein’s usefulness as an astrocyte biomarker using the fully automated LUMIPULSE^®^ system. Diagnostics.

[27] Bhusal A., Rahman M.H., Lee W.H., Lee I.K., Suk K. (2021). Satellite glia as a critical component of diabetic neuropathy: Role of lipocalin-2 and pyruvate dehydrogenase kinase-2 axis in the dorsal root ganglion. Glia.

[28] Lei Q., Jiang Z., Shao Y., Liu X., Li X. (2024). Stellate ganglion, inflammation, and arrhythmias: A new perspective on neuroimmune regulation. Front. Cardiovasc. Med..

[29] Thaung H.A., Baldi J.C., Wang H.-Y., Hughes G., Cook R.F., Bussey C.T., Sheard P.W., Bahn A., Jones P.P., Schwenke D.O. (2015). Increased efferent cardiac sympathetic nerve activity and defective intrinsic heart rate regulation in type 2 diabetes. Diabetes.

[30] Jubaidi F.F., Zainalabidin S., Taib I.S., Abdul Hamid Z., Mohamad Anuar N.N., Jalil J., Mohd Nor N.A., Budin S.B. (2022). The role of PKC-MAPK signalling pathways in the development of hyperglycemia-induced cardiovascular complications. Int. J. Mol. Sci..

[31] Grassi G., Biffi A., Dell’Oro R., Trevano F.Q., Seravalle G., Corrao G., Perseghin G., Mancia G. (2020). Sympathetic neural abnormalities in type 1 and type 2 diabetes: A systematic review and meta-analysis. J. Hypertens..

[32] Heusser K., Tank J., Diedrich A., Fischer A., Heise T., Jordan J. (2023). Limited evidence for sympathetic neural overactivation in older patients with type 2 diabetes mellitus. Front. Neurosci..

[33] Williams S., Raheim S.A., Khan M.I., Rubab U., Kanagala P., Zhao S.S., Marshall A., Brown E., Alam U. (2022). Cardiac autonomic neuropathy in type 1 and 2 diabetes: Epidemiology, pathophysiology, and management. Clin. Ther..

[34] Vinik A.I., Ziegler D. (2007). Diabetic cardiovascular autonomic neuropathy. Circulation.

[35] Lathrop D.A., Spooner P.M. (2001). On the neural connection. J. Cardiovasc. Electrophysiol..

[36] Verrier R.L., Antzelevitch C. (2004). Autonomic aspects of arrhythmogenesis: The enduring and the new. Curr. Opin. Cardiol..

[37] Thomas G.D. (2011). Neural control of the circulation. Adv. Physiol. Educ..

[38] Cuevas J. (2014). Molecular mechanisms of dysautonomia during heart failure. Focus on “Heart failure-induced changes of voltage-gated Ca^2+^ channels and cell excitability in rat cardiac postganglionic neurons”. Am. J. Physiol.-Cell Physiol..

[39] Ivanova N., Hristov M., Gateva P. (2025). Rodent models of diabetic neuropathy, role of calcium homeostasis in pain and KB-R7943 as a potential therapeutic. Int. J. Mol. Sci..

[40] Wu J., Hu H., Li X. (2025). Spinal neuron-glial crosstalk and ion channel dysregulation in diabetic neuropathic pain. Front. Immunol..

[41] Alles S.R., Smith P.A. (2021). Peripheral voltage-gated cation channels in neuropathic pain and their potential as therapeutic targets. Front. Pain Res..

[42] Hanani M., Spray D.C. (2020). Emerging importance of satellite glia in nervous system function and dysfunction. Nat. Rev. Neurosci..

[43] Li Y.-L., Li Y., Tu H., Evans A.J., Patel T.A., Zheng H., Patel K.P. (2025). Stellate Ganglia: A Key Therapeutic Target for Malignant Ventricular Arrhythmia in Heart Disease. Circ. Res..

[44] Birren S.J., Goodrich L.V., Segal R.A. (2025). Satellite glial cells: No longer the most overlooked glia. Cold Spring Harb. Perspect. Biol..

[45] Andreeva D., Murashova L., Burzak N., Dyachuk V. (2022). Satellite Glial Cells: Morphology, functional heterogeneity, and role in pain. Front. Cell. Neurosci..

[46] Qiu X., Yang Y., Da X., Wang Y., Chen Z., Xu C. (2024). Satellite glial cells in sensory ganglia play a wider role in chronic pain via multiple mechanisms. Neural Regen. Res..

[47] Souza G.R., Talbot J., Lotufo C.M., Cunha F.Q., Cunha T.M., Ferreira S.H. (2013). Fractalkine mediates inflammatory pain through activation of satellite glial cells. Proc. Natl. Acad. Sci. USA.

[48] Gonçalves N.P., Vægter C.B., Pallesen L.T. (2018). Peripheral glial cells in the development of diabetic neuropathy. Front. Neurol..

[49] Schmitt L.-I., Leo M., Kutritz A., Kleinschnitz C., Hagenacker T. (2020). Activation and functional modulation of satellite glial cells by oxaliplatin lead to hyperexcitability of sensory neurons in vitro. Mol. Cell. Neurosci..

[50] Zhou Z., Zhang H., Xiong H., Deng K.-Q., Zheng M., Zhang Y., Xu Z., Tian R., Zhang T., Kong X. (2025). Inhibition of Satellite Glial Cell Activation in Stellate Ganglia Prevents Ventricular Arrhythmogenesis and Remodeling After Myocardial Infarction. Circ. Arrhythm. Electrophysiol..

[51] Geraldes P., King G.L. (2010). Activation of protein kinase C isoforms and its impact on diabetic complications. Circ. Res..

[52] Yang Y., Zhao B., Wang Y., Lan H., Liu X., Hu Y., Cao P. (2025). Diabetic neuropathy: Cutting-edge research and future directions. Signal Transduct. Target. Ther..

[53] Eichberg J. (2002). Protein kinase C changes in diabetes: Is the concept relevant to neuropathy?. Int. Rev. Neurobiol..

[54] Gutiérrez A., Contreras C., Sánchez A., Prieto D. (2019). Role of phosphatidylinositol 3-kinase (PI3K), mitogen-activated protein kinase (MAPK), and protein kinase C (PKC) in calcium signaling pathways linked to the α1-adrenoceptor in resistance arteries. Front. Physiol..

[55] Lien C.-F., Chen S.-J., Tsai M.-C., Lin C.-S. (2021). Potential role of protein kinase C in the pathophysiology of diabetes-associated atherosclerosis. Front. Pharmacol..

[56] Wang F., Xu C., Reece E.A., Li X., Wu Y., Harman C., Yu J., Dong D., Wang C., Yang P. (2017). Protein kinase C-alpha suppresses autophagy and induces neural tube defects via miR-129-2 in diabetic pregnancy. Nat. Commun..

[57] Lo U., Selvaraj V., Plane J.M., Chechneva O.V., Otsu K., Deng W. (2014). p38α (MAPK14) critically regulates the immunological response and the production of specific cytokines and chemokines in astrocytes. Sci. Rep..

[58] Hall K.E., Liu J., Sima A.A., Wiley J.W. (2001). Impaired inhibitory G-protein function contributes to increased calcium currents in rats with diabetic neuropathy. J. Neurophysiol..

[59] Jagodic M.M., Pathirathna S., Nelson M.T., Mancuso S., Joksovic P.M., Rosenberg E.R., Bayliss D.A., Jevtovic-Todorovic V., Todorovic S.M. (2007). Cell-specific alterations of T-type calcium current in painful diabetic neuropathy enhance excitability of sensory neurons. J. Neurosci..

[60] Gallego M., Zayas-Arrabal J., Alquiza A., Apellaniz B., Casis O. (2021). Electrical features of the diabetic myocardium. Arrhythmic and cardiovascular safety considerations in diabetes. Front. Pharmacol..

[61] de Queiroz T., Lakkappa N., Lazartigues E. (2020). ADAM17-mediated shedding of inflammatory cytokines in hypertension. Front. Pharmacol..

[62] Wang K., Xuan Z., Liu X., Zheng M., Yang C., Wang H. (2022). Immunomodulatory role of metalloproteinase ADAM17 in tumor development. Front. Immunol..

[63] Vidal P., Lemmens E., Avila A., Vangansewinkel T., Chalaris A., Rose-John S., Hendrix S. (2013). ADAM17 is a survival factor for microglial cells in vitro and in vivo after spinal cord injury in mice. Cell Death Dis..

[64] Chen X., Yao J., Lai J., Lin L., Chen Y., Lin Y., Fang W., Ding C., Kang D. (2023). ADAM17 aggravates the inflammatory response by modulating microglia polarization through the TGF-β1/smad pathway following experimental traumatic brain injury. J. Neurotrauma.

[65] Dhir S., Derue H., Ribeiro-da-Silva A. (2024). Temporal changes of spinal microglia in murine models of neuropathic pain: A scoping review. Front. Immunol..

[66] Nemoto W., Yamagata R., Nakagawasai O., Hoshi T., Kobayashi R., Watanabe M., Tan-No K. (2025). Spinal ADAM17 contributes to the pathogenesis of painful diabetic neuropathy in leptin receptor-deficient mice. Biochem. Pharmacol..

[67] Bao X., Altenberg G.A., Reuss L. (2004). Mechanism of regulation of the gap junction protein connexin 43 by protein kinase C-mediated phosphorylation. Am. J. Physiol.-Cell Physiol..

[68] Hanani M., Blum E., Liu S., Peng L., Liang S. (2014). Satellite glial cells in dorsal root ganglia are activated in streptozotocin-treated rodents. J. Cell. Mol. Med..

[69] Tu H., Liu J., Zhang D., Zheng H., Patel K.P., Cornish K.G., Wang W.-Z., Muelleman R.L., Li Y.-L. (2014). Heart failure-induced changes of voltage-gated Ca2+ channels and cell excitability in rat cardiac postganglionic neurons. Am. J. Physiol.-Cell Physiol..

[70] Zhang D., Hu W., Tu H., Hackfort B.T., Duan B., Xiong W., Wadman M.C., Li Y.-L. (2021). Macrophage depletion in stellate ganglia alleviates cardiac sympathetic overactivation and ventricular arrhythmogenesis by attenuating neuroinflammation in heart failure. Basic Res. Cardiol..

[71] Zhang D., Tu H., Wang C., Cao L., Hu W., Hackfort B.T., Muelleman R.L., Wadman M.C., Li Y.-L. (2021). Inhibition of N-type calcium channels in cardiac sympathetic neurons attenuates ventricular arrhythmogenesis in heart failure. Cardiovasc. Res..

[72] Zhang D., Liu J., Tu H., Muelleman R.L., Cornish K.G., Li Y.-L. (2014). In vivo transfection of manganese superoxide dismutase gene or nuclear factor κB shRNA in nodose ganglia improves aortic baroreceptor function in heart failure rats. Hypertension.

