# Supplementary material for: Impact of PKC-MAPK Signaling on Cardiac Sympathetic Overactivation in Type-2 Diabetes Mellitus
Source: Int J Mol Sci. 2026 Jan 10;27(2):723. doi: 10.3390/ijms27020723 (PMC12840624; doi:10.3390/ijms27020723)
Supplement: Supplementary file 1 [file ijms-27-00723-s001.zip › ijms-4072287-supplementary.pdf]

Supplementary Table S1. Metabolic characteristics of sham and T2DM rats

	Sham (n=28)	T2DM (n=28)
Body weight (g)	385.07 ± 11.62	390.25 ± 9.03 (p=0.68)
Fasting blood glucose (mg/dl)	91 ± 2.11	447 ± 12.68* (p=0.04)

Data presented as means ± SEM, $p<0.05$ vs. Sham.

Supplementary Table S2. Antibodies used for immunofluorescence staining, western blot.

Target	Abbreviation	Application	Dilution	Company	Catalog number
S100 calcium binding protein	S100	IF	1:200	Agilent Dako	GA504
Tyrosine Hydroxylase	TH	IF	1:200	Sigma	T2928
Glial fibril acidic protein	GFAP	IF	1:200	Sigma	G3893
Phospho- Mitogen Activated Phosphokinase 14	p-MAPK14	Western	1:1000	Abcam	ab4822
Phospho- Protein Kinase C alpha	p- PKC-α	Western	1:1000	Thermofisher Scientific	PA5-118745
Protein Kinase C alpha	PKC-α	Western	1:1000	Abcam	ab32376
Mitogen Activated Phosphokinase 14	MAPK14	Western	1:1000	Abcam	ab170099
TNF-alpha converting enzyme (TACE)	ADAM17	Western	1:500	Abcam	ab39162
Connexin 43	Cx43	Western	1:500	Santa Cruz	sc-271837
βeta actin	β actin	Western	1:1000	Santa Cruz	sc-47778

Supplementary Table S3. List of primer used for q-PCR

**Gene Name**		
Connexin 43 (Cx43)	Forward<br>Reverse	GCG GCT TGC TGA GAA CCT AC<br>CAG TGG TGG CGT GGT AAG GA
PKC-alpha (PKC-α)	Forward<br>Reverse	GGAACTCAGGCAGAAGTTCG<br>CAGTTCCTCTGTTCCCTTCC
MAPK-14	Forward<br>Reverse	CCGAGCGATACCAGAACCT<br>CTTCACTGCCACACGATGTC
ADAM17 (TACE)	Forward<br>Reverse	GTGCTGACACCGACAACTCGT<br>CAGCTGGTCAATGAAATCCCAAA
